# Rizoma: a new comprehensive database on traditional uses of Chilean native plants

**DOI:** 10.3897/BDJ.10.e80002

**Published:** 2022-03-04

**Authors:** Sebastián Cordero, Lucía Abello, Francisca Gálvez

**Affiliations:** 1 Rizoma, Centro de Estudios Agroecológicos y Botánicos, Valparaíso, Chile Rizoma, Centro de Estudios Agroecológicos y Botánicos Valparaíso Chile

**Keywords:** Chile, ethnobotanical dataset, traditional knowledge, useful plants, wild edible plants, wild medicinal plants

## Abstract

**Background:**

We describe Rizoma, a new comprehensive online database on traditional uses of Chilean flora. The Rizoma database was built by reviewing multiple data sources on the uses of native plants and integrating phytogeographic and ecological aspects of plant species. This database attempts to safeguard traditional knowledge by making it available and visible to society, providing 1380 use records from 736 vascular plant species native to Chile. In addition, it contributes to a better understanding of the use patterns of Chilean native plants.

**New information:**

The Rizoma database includes 1380 use records from 736 vascular plant species native to Chile, representing 399 genera and 128 families. Each species record provides information on geographic distribution, phytogeographic origin, life form, life span and use category. In addition, the online version includes information on the mode of use of each species, as well as common names and photographs. The database serves as a traditional knowledge repository that contributes to preserving local biological and cultural diversity for future generations.

## Introduction

Since ancient times, humans have used wild plants for multiple purposes. Even today, many cultures worldwide still maintain the tradition of gathering wild plants due to their relevance to human survival and well-being (Toledo et al. 2009, McCarter and Gavin 2015). However, despite its importance, traditional knowledge on the use of plants is progressively eroding due to several sociocultural and ecological processes, such as economic globalisation, cultural homogenisation and environmental degradation (Cordero et al. 2020b). Nowadays, old traditions of plant gathering are being lost in most countries (Łuczaj et al. 2012); thus, it is crucial to increase efforts to safeguard traditional knowledge and preserve biological and cultural diversity for future generations (Asfaw 2009, Cordero et al. 2020a). Under an accelerated human-induced species loss scenario, traditional knowledge plays a critical role in biodiversity conservation. People protect useful plant species because they are essential elements within their cultures or religions (Susanti and Zuhud 2019). In addition, traditional knowledge provides insights for developing biodiversity conservation strategies, based on the observation and experience of local communities (Sutherland et al. 2013).

Ethnobotanical research is key to documenting knowledge about the use of wild plants and has increased over the past decades, with large inventories of useful plants published for several geographic regions (e.g. Tardío et al. 2006, Lentini and Venza 2007, Lulekal et al. 2011, Simkova and Polesny 2015). Moreover, some databases have been developed by compiling and systematising ethnobotanical data, with the aim not only to safeguard knowledge on useful plants, but also to promote their use, facilitating access to this kind of information to the non-scientific community (e.g. Loub et al. 2002, Ningthoujam et al. 2012, Noe 2019, Souza and Hawkins 2020). The availability and the visibility of ethnobotanical data are critical for traditional knowledge acquisition and maintenance because they increase the interest in using wild plants in modern societies (Simkova and Polesny 2015). Although knowledge is acquired mainly through parents and community members (Turreira-García et al. 2015), other less traditional sources of information have also been identified. Online resources, such as digital books and websites, are essential for knowledge acquisition, especially in urban contexts, where interaction with nature is limited by multiple factors (Cordero et al. 2020b). In a highly globalised world, easy access to ethnobotanical data through public websites is an alternative that has been scarcely explored, but could revitalise local identity and traditions (Menendez-Baceta et al. 2011).

Useful plant inventories and databases have not received sufficient attention in some countries. This is the case for Chile (a South American nation), where ethnobotanical data remains fragmented and difficult to access. Therefore, to promote the use, conservation and appreciation of Chilean native flora, we developed Rizoma, a comprehensive online database on the uses of wild plants. The Rizoma database contains information on the mode of use, use category, geographic distribution, phytogeographic origin, life form, life span, common names, taxonomic aspects and photographs.

## General description

### Purpose

Our primary goal is to provide a comprehensive database that allows easy and free access to traditional knowledge on the use of the Chilean native plants, contributing to its maintenance and appreciation, while avoiding its progressive erosion.

## Project description

### Title

Rizoma: a new comprehensive database on traditional uses of Chilean native plants.

### Study area description

Chile, South America.

## Sampling methods

### Sampling description

We collected the available ethnobotanical data through three steps of the literature review. First, we searched articles by using the Web of Science database (from January 1983 to December 2018), with the keywords "ethnobotan*", "ethnomedicin*", "ethnopharmacolog*", "gathering practice", "traditional knowledge", "traditional practices", "traditional plant uses", "detergent plants", "dye plants", "edible plants", "fodder", "magic* plants", "medicin* plants", "ritual plants", "veterinary plants", "cosmetic plants", "*craft* plants", "fuel plants", "psychotropic plants", "wood* plants", "construction plants", "non-timber forest products", "wild plant uses" + "Chile" in both English and Spanish following the PRISMA statement (Moher et al. 2009). This initial search returned 743 articles, which were refined by categories; engineering, meteorology, atmospheric sciences, soil science and others were considered irrelevant and excluded. From this, we obtained 222 articles, to which we applied a new filter by selecting only articles and reviews (i.e. removing proceedings papers, meeting abstracts), resulting in 217 articles. Then, we examined these articles by looking for abstracts that match the main criteria for providing information on the uses of Chilean flora. Based on the abstract selection, we considered 72 papers for full-article review. Finally, 62 articles were selected, focused on the uses of Chilean flora mainly from ethnobotanical, ethnographic, archaeobotanical and anthropological approaches.

In a second step, we repeated the search in Spanish through Google Scholar by using the same search keywords. We conducted this new search due to the scarcity of results returned by Web of Science using Spanish keywords. Several studies on the uses of Chilean flora have been published in local journals or bulletins not included in the Web of Science databases, limiting potentially valuable results. This initial search returned 515 results, but provided many spurious results. Subsequently, we filtered them by looking for abstracts (or descriptions according to availability), selecting 54 results. From this, we selected 35 after a full review. Lastly, in a third step, we reviewed monographs, theses and books related to the uses of Chilean flora available in thirteen university and municipal libraries, obtaining 171 documents. These three literature review steps resulted in 268 selected references listed in Suppl. material 1.

### Step description

The database only considers native wild plant species; thus, we excluded those alien or cultivated, based on the Database of alien plant species in Chile (Fuentes et al. 2012). We followed the Catalogue of the vascular plants of Chile (Rodriguez et al. 2018) to compare and update the scientific names, genera and families of the useful species and to remove duplicate synonymised species since this catalogue offers the most up-to-date taxonomic treatment for the Chilean flora. We also included common names (in Spanish) obtained from the reviewed literature. However, we only considered common names, provided together with scientific names to avoid any uncertainty about the identity of the species. Then, we determined the life form and life span for each species according to Rodriguez et al. (2018), as well as their geographical distribution (administrative regions of Chile) and phytogeographic origin (native or endemic). Although the endemic category represents a subset of the native category, it provides different information, highlighting that a great proportion of the useful Chilean flora only exists in one place of the world.

Finally, plants were grouped into 14 use categories according to the mode they are used as follows: construction (plants that serve as raw material for home construction), cosmetic (plants used for skin and hair care, as well as to maintain personal hygiene), detergent (plants that contain substances capable of removing fats or organic materials), dye (plants from which natural dyes are obtained for textile application), veterinary (plants used to treat diseases or conditions in domestic animals), edible (plants used for human consumption), fodder (plants consumed by domestic animals, mainly cattle), fuel (plants used to start and maintain fire for heating purposes), handicraft (plants that serve as raw material for the production of objects or products), magic-religious (plants used in incense, witchcraft, blessings and curses, as well as those with symbolic and religious value), medicinal (plants used to treat diseases, conditions and injuries in humans), psychotropic (plants that induce altered states of consciousness), woody (plants used for the construction of buildings, transportation, furniture and other elements) and others (includes those uses that do not match the categories described above; for example, tannery, hunting tools, mordants).

## Geographic coverage

### Description

**Figure 1.**
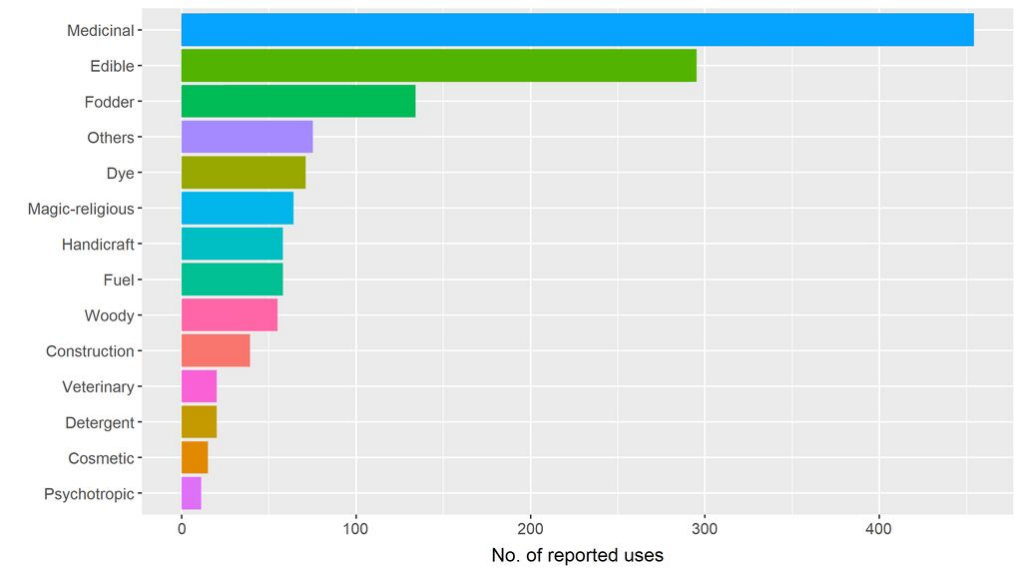
Number of reports for each of the 14 use categories, ordered from highest to lowest values.

**Figure 2.**
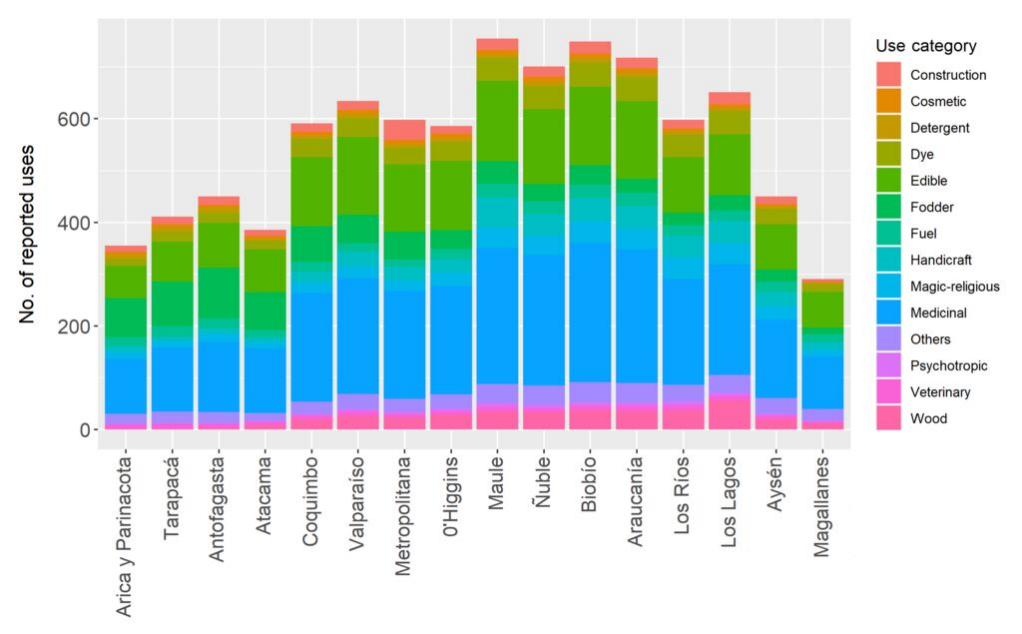
Number of reports by use categories for each administrative region of Chile.

Data were collected for the sixteen administrative regions of Chile (South America), with the highest number of useful species reported for south-central Chile: Maule (376 species), Biobío (375), Valparaíso (359) and Araucanía (357) Regions. The use categories that reported the largest number of records were medicinal, edible and fodder, both at the species level (Fig. 1) and the administrative regions of Chile (Fig. 2). Although using administrative regions to describe geographic coverage restricts statistical analyses, Fig. 2 provides a general overview of the geographic distribution of useful plants in Chile. Herbarium data are currently being collected to accurately assess geographic distribution patterns of useful flora in Chile and will be included in future database updates.

### Coordinates

-17.50 and -55.98 Latitude; -71.32 and -73.52 Longitude.

## Taxonomic coverage

### Description

The database includes 1380 use records from 736 vascular plant species native to Chile, belonging to 399 genera from 128 families (Suppl. material 2). The most species-rich families are Asteraceae (120 species), Fabaceae (46), Poaceae (41), Apiaceae (28), Solanaceae (23) and Cactaceae (22) (Table 1). The genera containing the highest number of useful species are *Adesmia* (18 species), *Baccharis* (12), *Azorella* (11), *Senecio* (11) and *Berberis* (10) (Table 2). According to our database, the species having the greatest number of uses are *Aristoteliachilensis* (Molina) Stuntz (8 records), *Azorellacompacta* Phil. (8), *Chusqueaquila* Kunth (7), *Gevuinaavellana* Molina (7), *Laureliasempervirens* (Ruiz & Pav.) Tul. (7), *Nothofagusobliqua* (Mirb.) Oerst. (7), *Prosopischilensis* (Molina) Stuntz emend. Burkart (7) and *Tessariaabsinthioides* (Hook. & Arn.) DC. (7) (Table 3). In the online version, photographs are currently provided for 340 species, although this aspect is continuously developing.

## Usage licence

### Usage licence

Creative Commons Public Domain Waiver (CC-Zero)

## Data resources

### Data package title

Rizoma: a new comprehensive database on traditional uses of Chilean native plants.

### Resource link

https://ceab-rizoma.com/database/

### Number of data sets

1

### Data set 1.

#### Data set name

Traditional uses of Chilean native plants

#### Number of columns

21

#### Description

Traditional uses of Chilean native plants containing information on the mode of use, geographic distribution, phytogeographic origin, life form, life span and taxonomic data (Suppl. material 2).

**Data set 1.** 

Column label	Column description
Family	The scientific name of the family in which the taxon is classified.
Genus	The scientific name of the genus in which the taxon is classified.
ScientificName	The full scientific name of the species.
Origin	Phytogeographic origin of the species ("native"; "endemic").
Distribution	Geographical area where the species occurrs (administrative regions of Chile: "ayp" = Región de Arica y Parinacota"; "ant" = Región de Antofagasta; "tar" = Región de Tarapacá; "ata" = Región de Atacama; "coq" = Región de Coquimbo; "val" = Región de Valparaíso; "rm" = Región Metropolitana de Santiago; "lgo" = Región del Libertador General Bernardo O'Higgins; "mau" = Región del Maule; "nub" = Región de Ñuble; "bio" = Región del Biobío; "ara" = Región de La Araucanía; "lri" = Región de Los Ríos; "lla" = Región de Los Lagos; "ays" = Región de Aysén del General Carlos Ibáñez del Campo; "mag" = Región de Magallanes y de la Antártica Chilena").
LifeSpan	Plant growth form ("annual"; "biennial"; "perennial").
LifeForm	Seasonal growth cycle ("tree"; "succulent tree"; "subshrub"; "epiphytic subshrub"; "parasitic subshrub"; "succulent subshrub"; "climbing subshrub"; "shrub"; "parasitic shrub"; "succulent shrub"; "climbing shrub"; "herb"; "aquatic herb"; "epiphytic herb"; "parasitic herb"; "climbing herb").
Construction	Plants used as raw materials for home construction.
Cosmetic	Plants used for skin and hair care and to maintain personal hygiene.
Detergent	Plants used to remove fats or organic materials.
Dye	Plants used to obtain natural dyes for textile application.
Edible	Plants used for human consumption.
Fodder	Plants consumed by domestic animals.
Fuel	Plants used to start and maintain fire for heating purposes.
Handicraft	Plants used as raw materials to produce objects or products.
Magic-religious	Plants used for blessings and curses or symbolic-religious value.
Medicinal	Plants used to treat medical conditions in humans.
Psychotropic	Plants used to induce altered states of consciousness.
Veterinary	Plants used to treat diseases or conditions in domestic animals.
Woody	Plants used for the construction of buildings, transportation, furniture, and other elements.
Others	Includes those uses that do not match other categories.

## Additional information

### Availability

The database has now been publicly released on the website of the Centro de Estudios Agroecológicos y Botánicos Rizoma (https://ceab-rizoma.com/database/), where data can be visualised. A search engine has been included that allows to search results using category filters in addition to a simple search system.

## Supplementary Material

0934D541-CA22-5D30-8E70-D4423B4F8229 10.3897/BDJ.10.e80002.suppl1 Supplementary material 1 Reviewed references Data type References Brief description The reviewed reference list containing information on the use of Chilean plants for 736 native species. File: oo_629401.csv https://binary.pensoft.net/file/629401 Sebastián Cordero, Francisca Gálvez & Lucía Abello

32147A2E-F18C-558E-BE5E-23DD9B8DADC6 10.3897/BDJ.10.e80002.suppl2 Supplementary material 2 Traditional uses of the Chilean native plants Data type Species list Brief description Collected data on the uses of Chilean native plants, containing 1380 use records for 736 vascular plant species, distributed in 399 genera and 128 families. The records of each species provide data on geographic distribution, phytogeographic origin, life form, life span, mode of use and use category. File: oo_645329.csv https://binary.pensoft.net/file/645329 Sebastián Cordero, Francisca Gálvez & Lucía Abello

## Figures and Tables

**Table 1.** The top 20 families with the most useful species ranked from highest to lowest value, including the total number of use records for each.

Rank	Family	No. of species	No. of use records
1	Asteraceae	120	221
2	Fabaceae	46	81
3	Poaceae	41	74
4	Apiaceae	28	46
5	Solanaceae	23	54
6	Cactaceae	22	34
7	Rosaceae	16	29
8	Cyperaceae	14	20
9	Myrtaceae	12	33
10	Verbenaceae	11	24
11	Juncaceae	11	22
12	Plantaginaceae	11	15
13	Nothofagaceae	10	29
14	Berberidaceae	10	28
15	Boraginaceae	10	17
16	Pteridaceae	10	10
17	Chenopodiaceae	9	19
18	Malvaceae	9	15
19	Oxalidaceae	9	17
20	Ericaceae	8	17

**Table 2.** The top 20 genera with the most useful species ranked from highest to lowest value, including the total number of use records for each.

Rank	Genus	No. of species	No. of use records
1	*Adesmia*	18	29
2	*Baccharis*	12	34
3	*Azorella*	11	20
4	*Senecio*	11	19
5	*Nothofagus*	10	29
6	*Berberis*	10	28
7	*Oxalis*	9	17
8	*Haplopappus*	9	11
9	*Juncus*	8	16
10	*Solanum*	8	10
11	*Gaultheria*	7	13
12	*Echinopsis*	7	10
13	*Valeriana*	7	10
14	*Dioscorea*	7	7
15	*Fabiana*	6	22
16	*Alstroemeria*	6	10
17	*Acaena*	6	9
1	*Tropaeolum*	6	7
19	*Schinus*	5	13
20	*Festuca*	5	8

**Table 3.** The top 20 species with the highest number of traditional uses.

Rank	Scientific name	No. of uses
1	*Aristoteliachilensis* (Molina) Stuntz	8
2	*Azorellacompacta* Phil.	8
3	*Chusqueaquila* Kunth	7
4	*Gevuinaavellana* Molina	7
5	*Laureliasempervirens* (Ruiz & Pav.) Tul.	7
6	*Nothofagusobliqua* (Mirb.) Oerst.	7
7	*Prosopischilensis* (Molina) Stuntz emend. Burkart	7
8	*Tessariaabsinthioides* (Hook. & Arn.) DC.	7
9	*Araucariaaraucana* (Molina) K. Koch	6
10	*Jubaeachilensis* (Molina) Baill.	6
11	*Bacchariscalliprinos* Griseb.	6
12	*Berberismicrophylla* G. Forst.	6
13	*Cryptocaryaalba* (Molina) Looser	6
14	*Embothriumcoccineum* J.R. Forst. & G. Forst.	6
15	*Fabianasquamata* Phil.	6
16	*Luzuriagaradicans* Ruiz & Pav.	6
17	*Peumusboldus* Molina	6
18	*Baccharisalnifolia* Meyen & Walp.	5
19	*Baccharisboliviensis* (Wedd.) Cabrera	5
20	*Baccharistola* Phil.	5
